# Characterization of Back-Scattering and Multipath in a Suburban Area after the Calibration of an X-Band Commercial Radar

**DOI:** 10.3390/s20020463

**Published:** 2020-01-14

**Authors:** Gaspare Galati, Gabriele Pavan, Christoph Wasserzier

**Affiliations:** 1 Department of Electronic Engineering, Tor Vergata University of Rome and CNIT—National Inter-University Consortium for Telecommunications, RU of Rome, via del Politecnico 1, 00133 Rome, Italy; gaspare.galati@uniroma2.it; 2 Fraunhofer Institute for High Frequency Physics and Radar Techniques FHR, Fraunhoferstrasse 20, 53343 Wachtberg, Germany; christoph.wasserzier@fhr.fraunhofer.de or

**Keywords:** radar cross-sections, multipath, radar calibration

## Abstract

The increasing interest in the radar detection of low-elevation and small-size targets in complicated ground environments (such as urban, suburban, and mixed country areas) calls for a precise quantification of the radar detection capabilities in those areas. Hence, a set of procedures is devised and tested, both theoretically and experimentally, using a commercial X-band radar, to (i) calibrate the radar sensor (with an online evaluation of its losses) using standard scatterers, (ii) measure the multipath effect and compensate for it, and (iii) create “true radar cross section” maps of the area of interest for both point and distributed clutter. The above methods and the related field results are aimed at future qualification procedures and practical usage of small, cheap, and easily moveable radars for the detection of low-observable air targets, such as unmanned air vehicles/systems (UAV/UAS), in difficult ground areas. A significant set of experimental results as discussed in the paper confirms the great relevance of multipath in ground-based radar detection, with the need for correcting measures.

## 1. Introduction

Unwanted echoes represent a significant problem in many radar applications, such as the surveillance of airports, harbors, and inhabited areas. These echoes, globally referred to as “clutter” [1], can be generated by both continuous (i.e., distributed) or discrete sets of scatterers. Discrete sources of clutter are, for example, road/rail infrastructures, buildings, water towers, and large lampposts. On the other hand, grass, vegetation, terrain, etc. represent distributed clutter sources, whose echoes are measured as the radar cross section per unit area. Clutter may be orders of magnitude more intense than the useful targets of interest, strongly affecting their visibility. Moreover, the dynamic range of the clutter echoes and their probability distribution widely vary both in time (at a given resolution cell) and in space (i.e., from adjacent radar resolution cells at the same time). These effects strongly depend on the radar installation in the operational area.

Ground clutter models are widely studied, see for instance [1,2,3], but, in spite of the available literature and databases, dedicated measurements are needed in order to obtain the clutter levels in a given environment, especially when both discrete and distributed sources are present.

In order to generate valuable clutter maps, it is necessary to operate with a well calibrated radar sensor, that is, setting of a precise relationship between the received power and the radar cross section (RCS) in each resolution cell. The classical way to calibrate a radar sensor by measuring the various terms that made up the radar equation [4] (i.e., antenna gain, transmitted power, losses, etc.) may be lengthy and cumbersome. In the literature [5,6], a more cost/effective approach is investigated based on reconstructing the relationship between the RCS of known, standard objects (metallic spheres and corner reflectors) and the received power. This relationship is a linear law above the noise plafond and below the receiver saturation zone.

After calibration, realistic clutter maps can be generated [7,8,9,10]. They may be used, for example, to design the siting of X-band surveillance radars [11] for the surveillance of airports (surface movements radar and apron control) and for the detection of unauthorized unmanned aerial vehicles (UAVs also known as drones) in urban and suburban areas. The possibility for the UAVs to carry on payloads makes their use both very interesting and potentially dangerous. The Federal Aviation Administration (FAA) regulations in the USA regarding the use of drones in commercial applications are clearly defined, while hobby or recreation usages are encouraged to follow the safety guidelines [12]. The problem of drones’ control over a limited area is strongly rising up. The first idea is to, in some way, reproduce the surveillance system in Air Traffic Control (ATC) which uses dedicated radar sensors. However, they are very expensive and not easily deployable: typically, operating at S-band (2–3 GHz), these radars are more suitable for detecting big objects, like an airplane, rather than small drones. On the other hand, drones might be detected by cheaper commercial X-band (9.2–9.5 GHz) marine radars, either with a “solid state” transmitter or with a magnetron transmitter, which are very portable and easily deployable as compared with the large ATC surveillance radars.

The RCS of drones has recently been investigated. In [13], the numerical and experimental results are shown for a quadcopter drone (DJI Phantom2) at 10 GHz. The measurements of drones, carried out in an anechoic chamber, have shown RCS variations from −30 dBm^2^ to −10 dBm^2^. In [14], experimental results, at a range of up to 2 km, with an average altitude (above ground level) of 30 m, are reported using a commercial drone (DJI-Phantom4) and a ubiquitous FMCW radar system working at 8.75 GHz. The authors concluded that the drone echoes show the statistical behavior of Swerling I targets with an average RCS close to −20 dBm^2^. In [15], the radar signals in both the range and Doppler domains are analyzed for a quadcopter drone (DJI Phantom2), as well as three different birds (Hooded Vulture, Eurasian Eagle Owl, and Barn Owl). The authors, using an S-band coherent multistatic radar, found that the micro-drone signatures were comparable (in RCS) to those of birds. In [16], X-band measurements of flying sea birds have been carried out in order to investigate the potential of extracting the features for target recognition from micro-Doppler signatures.

In spring 2017, at Tor Vergata University in Rome, the preliminary experimental results were obtained using a commercial X-band marine radar to detect drones with the aim to compare the drone’s echo to those coming from known reflectors, in order to derive the radar visibility of the drone [17]. However, in this experimental research, it was soon clear that back-scattering and multipath, because of both continuous and discrete sources (often found in a suburban area), constitute a serious problem for small aircraft detection. The preliminary results, regarding the multipath analysis as a result of the land surface using an X-band radar, are shown in [18]. The relevant evaluations are based on the measured RCS variations of a calibrated target taken during the NATO SET-225 (Sensors and Electronic Technology Group-225) field trials in June 2018 at the Fraunhofer Institute FHR in Wachtberg, Germany.

During 2018 and in spring/summer 2019, more trials were carried out at Tor Vergata University. Some of the acquired data will be described in the following so as to highlight the problems related to the back-scattering and to the multipath in the frame of the detection of small targets.

This work is an extended version of the paper “Environmental Effects on Ground-based Radar Measurements” in Proceeding of the Sixth IEEE Workshop Metro Aerospace, Torino, Italy, June 2019 [19]. The paper is organized as follows. Section 2 recalls the system calibration procedure, including the estimation of the total radar losses from the antenna to the receiver. Section 3, considering the effects of terrain, describes the reflection coefficient and the multipath with its possible compensation. Section 4 and Section 5 show the evaluation of some aspects of multipath in the Tor Vergata area and in the Wachtberg area using calibrated targets. Using opportunity targets, in Section 6, the non-stationary nature (in time and in space) of the multipath is highlighted in a suburban environment. Final considerations and perspectives are reported in Section 7. Appendix A recalls the multipath.

## 2. Radar System Calibration and Estimation Losses

For research purposes, the Radar Business Unit (RBU) of the Navico Company has supplied Tor Vergata University of Rome, on loan to use, an International Maritime Organization (IMO) compliant marine radar (model Simrad SRT). The main parameters of this set are shown in Table 1.

**Figure 1:**
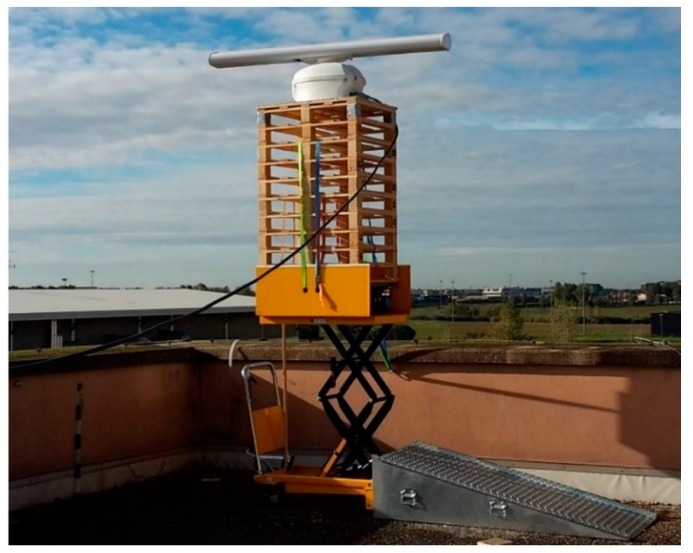
The Simrad/Navico SRT Radar and its lifter on the top of the Information Engineering building (Tor Vergata University).

Figure 1 shows the antenna and the Tx/Rx unit on the top of the Information Engineering building at Tor Vergata University in Rome. The radar is placed in the west corner of the upper terrace and is installed on a lifter capable of increasing the height of the radar antenna by up to 1 m. Arbitrary step positions are made possible for data acquisition, so as to evaluate the multipath effects, as explained in the following.

All of the trials shown in this paper have been performed in the short range mode (see Table 1) with a pulse length of 50 ns (bandwidth B = 20 MHz) and PRF = 3000 Hz. In this set-up, the power of the receiver noise $P_{noise}=Fk_{B}TB$ (F is the noise figure, $k_{B}$ is the Boltzmann’s constant, and T=290 K is the reference temperature) is equal to −96 dBm. The range resolution is 7.5 m, with a sampling interval of 3 m. Because of the horizontal beam-width of 1.35°, the nominal azimuth resolution is 12 m and 24 m at distances of 500 and 1000 m, respectively.

Downstream of the analog to digital conversion, for each antenna revolution, the acquired digital signals, coded in 256 levels, hereafter called $ADC_{level}$ (0–255), are recorded in a (Range, Azimuth) matrix with 2048 rows (range-bins), corresponding to a maximum range of 6144 m (3 m×2048), and a number of columns (azimuth-bins) nominally equal to 7500 at the rotating speed of 24 RPM and a PRF equal to 3000 Hz. The (Range, Azimuth) matrix allows us to display the plan position indicator (radar PPI).

The received power, expressed in dB above one milliwatt (dBm) is estimated by a linear relationship, as follows:

(1) $$P\left(\mathrm{dBm}\right)=\alpha \cdot ADC_{level}-\beta$$

where the constant α in (dB/level) is associated to each measured ADC level, while the constant β relates to the noise floor level ($P_{noise}$). These constants are obtained by radar calibration, without the need for dedicated measurements on the radar set.

The radar calibration is fully described in the literature [5]. The main steps are summarized as follows:

- Three octahedral corner reflectors (CRs) having scaled dimensions of the side a (large a=45 cm, medium a=22.5 cm, and small a=11.25 cm), and a coated sphere with a radius of r=20 cm, are put in a suitable position (characterized with terrain masking by suited natural fences) with both land clutter and multipath effects practically absent.
- Four independent measurements of the $ADC_{level}$, obtained by averaging eight measurements for each target, are recorded.
- The theoretical RCS (in ${\mathrm{dBm}}^{2}$) of CR’s are normalized to the RCS of the sphere.
- From step (ii), on the plane ($ADC_{level},RCS_{norm}$), four points are plotted.
- The slope of the regression line among these points estimates the parameter *α*, while the parameter β is estimated imposing on the straight line to pass on the mean noise point.

The measurements did confirm that in the chosen configuration, the multipath factor [4] is very close to the unit, thus allowing us to estimate, with an accuracy of the order of 1%, the parameters α and β of Equation (1). It resulted α=0.3792 (dBm/level) and β=105.48 (dBm). More details on this procedure are presented in [5].

The received power, $P_{r}$, is theoretically evaluated by the Radar Equation (2), including the overall system losses (L) up to the analog-to-digital-converter (ADC), as follows:

(2) $$P_{r}=\frac{P_{t}G^{2}\lambda^{2}}{{\left(4\pi \right)}^{3}}\cdot \frac{\sigma }{R^{4}}\cdot \frac{\eta^{4}}{L}$$

For a given distance, R, of the target, in the linear range of the receiver response, $P_{r}$ is proportional to the RCS of the target (σ) and to the ratio $\frac{\eta^{4}}{L}$, where L denotes the losses and $\eta^{4}$ is the contribution due to multipath, analyzed in Appendix A. Inverting Equation (2) and using Equation (1) for the $P_{r}$ expressed in dB, the RCS, $\sigma_{{\mathrm{dBm}}^{2}}=10\cdot log_{10}\left(\sigma \right)$, of a single target is written as follows:

(3) $$\sigma_{dBm^{2}}=\alpha \cdot ADC_{level}-\beta +L_{\mathrm{dB}}-\eta_{\mathrm{dB}}^{4}+4\cdot R_{\mathrm{dB}}-C_{\mathrm{dB}}$$

where $C_{\mathrm{dB}}=10\cdot log_{10}\left[\frac{P_{t}G^{2}\lambda^{2}}{{\left(4\pi \right)}^{3}}\right]$ includes the various radar parameters and constants. Using α=0.3792 (dBm/level) and β=105.48 (dBm; under the hypothesis that in operational conditions, such as the protection by a fence, $\eta_{dB}^{4}≅0$), the losses $L_{dB}$ in Equation (3) can be estimated as the value that minimizes the mean square error between the theoretical and the measured radar cross sections of the dedicated targets (four in this study). This resulted in $L_{dB}=-3.92\;\mathrm{dB}$. This value is compatible with the manufacturer data, kindly supplied by Navico RBU.

In general, it is not easy to evaluate, in one shot, radar losses with a residual error below 1 dB in typical conditions, and, to the best of the authors’ knowledge, these overall black-box measurements of the radar receiver and overall losses, using standard reflectors and a fence, are novel.

## 3. Evaluation of Multipath and of Reflection Coefficient

The previous discussion is related to those very particular conditions (e.g., rays blocked by a fence) in which the multipath on the vertical plane is negligible. Conversely, in the presence of a multipath [1], that is a normal situation in surface radars, the calibration by standard reflectors requires evaluating the multipath factor $\eta^{4}$ in the particular geometry and propagations conditions. The multipath factor is the ratio between the amplitude of the electro-magnetic field on the target and the amplitude in the (theoretical) case of the absence of multipath. This factor depends on the complex reflection coefficient of the surface $\Gamma =\rho \cdot e^{\psi_{r}}$, and on the phase shift $\psi_{\Delta }$, related to the path difference Δ between the direct and the reflected signals.

Following the developments and the notations in Appendix A, $\eta^{4}$ is expressed as follows:

(4) $$\eta^{4}={\left[1+2\rho \cdot \cos\left(\psi_{r}+\psi_{\Delta }\right)+\rho^{2}\right]}^{2}$$

For a given polarization, the parameters ρ and $\psi_{r}$ depend on the electromagnetic properties of the surface, that is, the real and imaginary part of its dielectric constant $\epsilon =\epsilon^{\prime }-j\epsilon^{\prime \prime }$, the frequency, the polarization, and on the grazing angle $\psi_{g}$. For the horizontal polarization, $\Gamma_{H}=\frac{\sin\left(\psi_{g}\right)-\sqrt{\epsilon -\cos^{2}\left(\psi_{g}\right)}}{\sin\left(\psi_{g}\right)+\sqrt{\epsilon -\cos^{2}\left(\psi_{g}\right)}}$.

In addition, considering the roughness of the surface, the global reflection coefficient is $\Gamma_{total}=\Gamma \cdot \rho_{rough}$, where $0<\rho_{rough}<1$ depend on the grazing angle, $\psi_{g}$, and on the r.m.s. roughness in the vertical direction $\sigma_{h}$.

**Figure 2:**
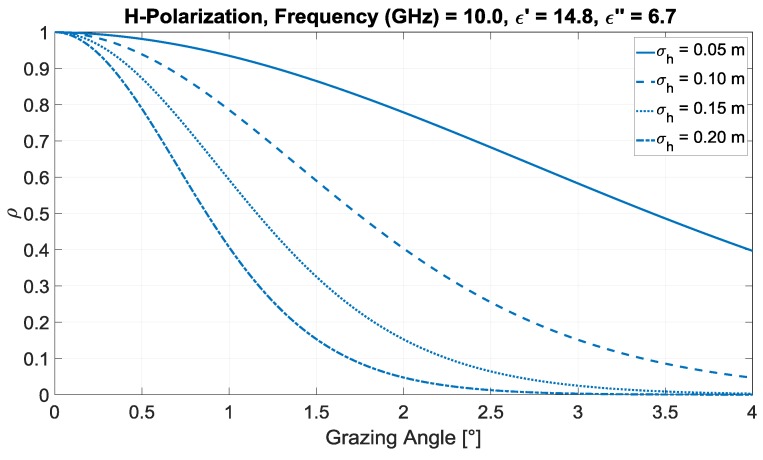
Modulus of the reflection coefficient ρ (X band, horizontal polarization) for soil (clay) with moisture of 35 g/cm^3^, low grazing angles, and four values of $\sigma_{h}$.

For flat soil (clay) with a moisture content of $35\;g/{\mathrm{cm}}^{3}$, we may use (for the horizontal polarization of the SRT) values of ϵ′=14.8 and ϵ″=6.7 (valid at 10.0 GHz [20]) as a good approximation of the 9.41 GHz data. Including the contribution of the land roughness ($\sigma_{h}$), Figure 2 shows the amplitude, ρ, of the reflection coefficient for $\sigma_{h}=0.05,\;010,\;0.15,\;\mathrm{and}\;0.20\;m$. For low grazing angles (in the order of 1 or 2 degrees), this coefficient is within 0.05<ρ<0.9.

In the horizontal polarization, the phase of the reflection coefficient for low grazing angles can be supposed to be equal to π, as follows: for grazing angles between 0.5° and 3°, its values range from 179.95° to 179.65° (H-polarization, f=10.0 GHz, soil moisture $35\;g/{\mathrm{cm}}^{3}$, ϵ′=14.8, and ϵ″=6.7), that is, Γ=−ρ, is not affected by the $\sigma_{h}$ variation. The roughness contribution to the reflection coefficient is real.

**Figure 3:**
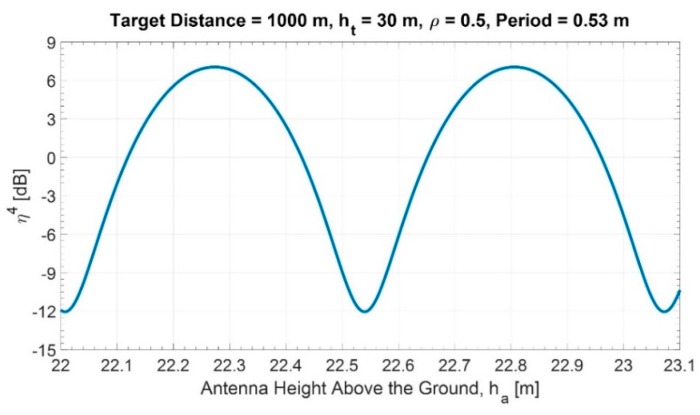
Multipath factor $\eta^{4}$ vs. the antenna height (target distance D=1000 m and target height of $h_{t}=30\;m$). The period $\frac{\lambda D}{2h_{t}}$ is equal to 0.53 m.

In Appendix A, Table A1 reports the maximum variation in the “apparent” RCS of the target due to multipath. For example, considering ρ≅0.5 (corresponding to 1.75° grazing angle and $\sigma_{h}=0.1$ m), a radar target distance D=1000 m and 30 m target height, Figure 3 shows a sample of the variation of the multipath factor with the radar antenna height, $h_{a}$, varying from 22.0 m to 23.1 m. The distance and the height of the target correspond to a certain target (lamppost L4, see Section 6) and the antenna height to the installation on the roof of the Information Engineering building at Tor Vergata University. The latter height can be controlled by varying the vertical radar position of the antenna by a mechanical elevator, as shown in Figure 1. With this set-up, we were able to reconstruct curves, such as in Figure 3, in order to estimate (with a best fit procedure) the modulus ρ of the reflection coefficient at the given experimental conditions.

From curves such as the one in Figure 3, the maximum value $\eta_{max}$ and the minimum value $\eta_{min}$ of the multipath factor are easily estimated. As $\eta_{max}$ is equal to 1+ρ, and $\eta_{min}$ is equal to 1−ρ, the modulus of the reflection coefficient is readily estimated, as follows:

(5) $$\rho =\frac{\eta_{max}-\eta_{min}}{\eta_{max}+\eta_{min}}$$

In practice, the effect of the multipath in the RCS measurements can be corrected, as described in the following procedure.

(a) Set the target under test (distance D from the radar and the height of its main scatter $h_{t}$).
(b) Evaluate the period of the factor $\eta^{4}$ versus the antenna height $h_{a}$. It is $\frac{\lambda D}{2h_{t}}$ (Appendix A).
(c) Define the step $\Delta h_{a}$ to obtain a significant number (e.g., ten) of positions inside the period.
(d) Record the received power, proportional to $\eta^{4}$, for each position, and draw the best-fit curve.
(e) Find the maximum and the minimum values of $\eta^{4}$.
(f) Use Equation (5) to obtain ρ.
(g) Use Equation (A7) to calibrate the received power.

## 4. Multipath Evaluation in the Tor Vergata Area

**Figure 4:**
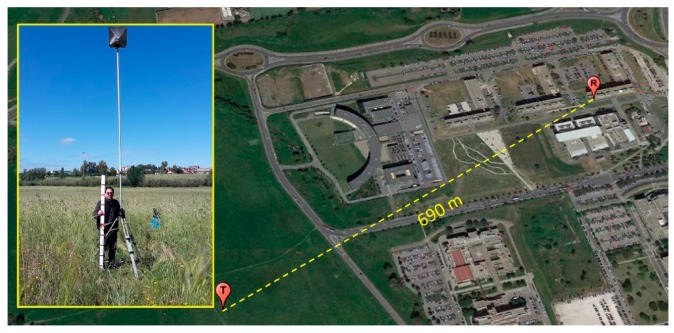
R and T denote the positions of the radar on the roof of the Information building (41°51′6.40″ N, 12°37′0.50″ E) and of the target (41°51′6.40″ N, 12°37′0.50″ E), respectively.

The first experiments were carried out in 2018 with a fixed radar installation for various heights of the same target (a corner reflector on the top of a pole, see left part of Figure 4).

Subsequently, in 2019, the radar elevator, shown in Figure 1, allowed us to vary the antenna height (in addition to the variation of the corner reflector height). The aim was to quantify the effect of the multipath on the RCS measurements, and to evaluate the reflection coefficient of the terrain (land with vegetation). For this goal, it was decided to keep the reflection point fixed on the ground, in order to be free from unwanted variations of the reflecting surface.

On 16 May 2019, a trihedral CR with an RCS of $7.2{\;\mathrm{dBm}}^{2}$ has been posed (as a target) on a tripod at a distance of 690 m from the radar (see Figure 4). The weather conditions were cloudy sky with sunshine, T=16 °C, and no wind.

Considering the starting position of the measurements (antenna height $h_{a}=23\;m$ and the target height $h_{t}=4.6\;m$), the reflection point on the ground is 575 m away from the radar. To keep the distance of the reflection point unchanged, we varied the height of the antenna and the target, and being $h_{a}/h_{t}=5$, it was necessary to vary the $h_{a}$ and $h_{t}$ (Equation (A1)) according to the values shown in Table 2. Considering the maximum ranges of elevation for the target and for the antenna, five positions were deemed sufficient.

Table 2 reports, for each position, the time of the acquisition and the measured RCS of the corner reflector. Remember that the theoretical value of the RCS of this CR is $7.2{\;\mathrm{dBm}}^{2}$.

**Figure 5:**
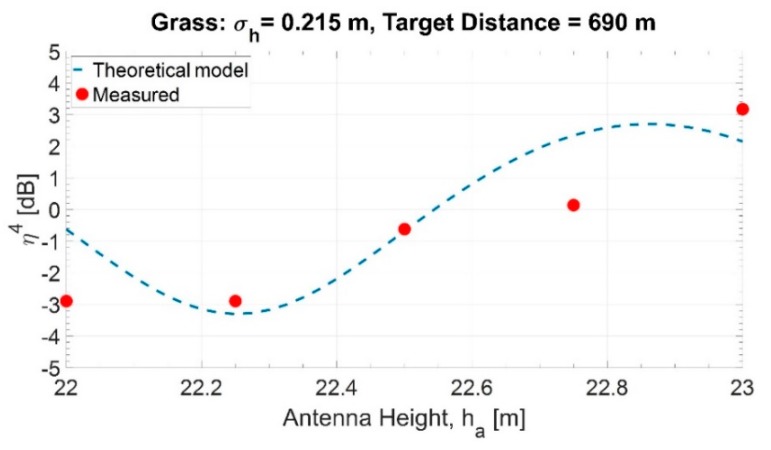
Theoretical and measured multipath factor with a constant position of the reflection point.

For the five positions, the mean grazing angle is 2.24°. Using Equation (1) with α=0.3792 and β=105.48, the received power has been evaluated. Hence, the multipath factor has been estimated using the average of the five received power values for each position. Using Equation (5), the amplitude of the reflection coefficient results in ρ=0.17. This value is compatible with vegetated land, with the height of the vegetation in the order of 10 to 15 cm, which is consistent with the observed height of the grass on the meadow. Figure 5 shows the theoretical multipath factor and the measured one.

The differences between the theoretical model and the measurements of this early experiment have been attributed to various unfavorable elements of the experimental area, including the following: (i) the land not being flat, (ii) the vegetation not being uniform, and (iii) the variation of the grazing angle versus $h_{a}$.

In a more regular environment, such as the one described in Section 5, the multipath analysis has shown a better agreement with the theory [19].

The ensuing experiments were performed, and their results are presented in the following. One experiment, conducted at Fraunhofer FHR, investigates the influence of variations in the vegetation on the multipath effect, as presented in Section 5. A second set of experiments, performed over a period of more than one month, and presented in Section 6, was conducted at Tor Vergata, aiming for an investigation of the temporal influences on the multipath effect.

## 5. Multipath Effects of Different Vegetations Observed in the Area of Wachtberg

Trials were conducted in June 2018, with a continuous emission (CE) noise radar at the Fraunhofer FHR Institute in Wachtberg, Germany. They addressed different aspects of noise radar, and were carried out by members of the NATO research task group (RTG) SET-225. The noise radar (NR) used during these experiments was developed by FHR, and is referred to as FHR-NR; it operates in the X-Band, more precisely in the standard maritime navigation band ranging from 9200 MHz to 9500 MHz, and thus, the same frequency band as the Simrad SRT radar, which allows for a direct comparison of the multipath effects observed in Tor Vergata with the ones at the FHR outpost in Wachtberg.

During the multipath trials, the operational signal bandwidth was B=50 MHz, corresponding to a range resolution not less than 3 m. The transmitted signal was a random waveform with a Gaussian distribution and less than 2 W of effective radiated power.

**Figure 6:**
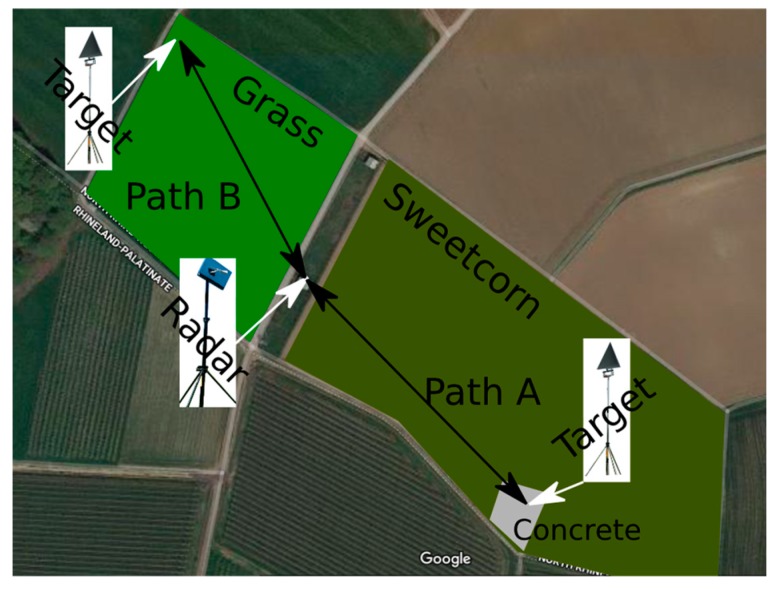
Illustration (from Google Earth) of the experimental setup in the fields of Wachtberg. Two different propagation paths were measured with the same radar and the same standard target. Sweetcorn was grown in the field of propagation path A (186 m), whereas grass was grown on the field of propagation in path B (159 m).

Dedicated experiments were conducted to characterize the effect of different kinds of vegetation on the multipath. Figure 6 shows the setup of the experiments with two positions of the same reference radar target. FHR-NR was installed inside a shelter at a small facility in Wachtberg, surrounded by fields. On one side of the shelter, there was a field of sweetcorn, with an approximate height of 2 m. On the other side, there was grassland of approximately 50 cm in height. The first position of the standard target was located in the sweetcorn field on a block of concrete at a 186 m distance to the radar (path A). The second target position had a radar distance of 159 m, and was located close to the end of the grass field (path B).

**Figure 7:**
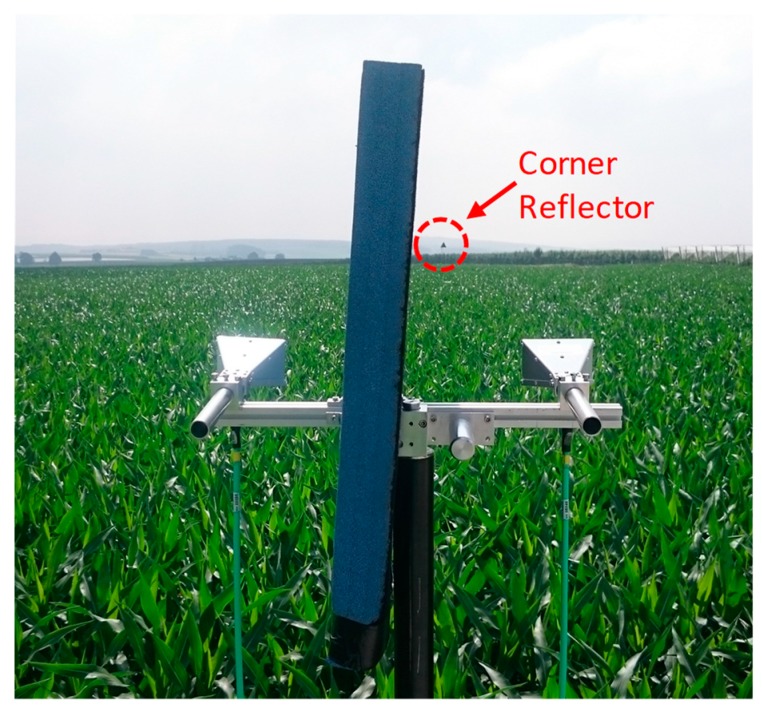
The two radar antennas were mounted on a tripod with variable heights. A plate of absorbing material was installed between the antennas for isolation purposes. This picture shows the propagation over sweetcorn along path A.

The standard target, used for both experiments, was a large trihedral corner reflector (CR) with a radar cross section not less than $1000{\;m}^{2}$. This corner reflector was mounted on a tripod, fully extended during all of the experiments at a constant height of 4.1 m. The height of the radar transmitting and receiving antenna system, made up by two standard horn antennas on a second tripod, as shown in Figure 7, varied in intervals of 10 cm from 2.6 m (path A), respectively 2.2 m (path B), up to a maximum height of 3.6 m.

A detailed analysis of this experiment can be found in [19]. The main points are summarized here and compared with the previously discussed results from the experiments performed at the Tor Vergata University of Rome.

**Figure 8:**
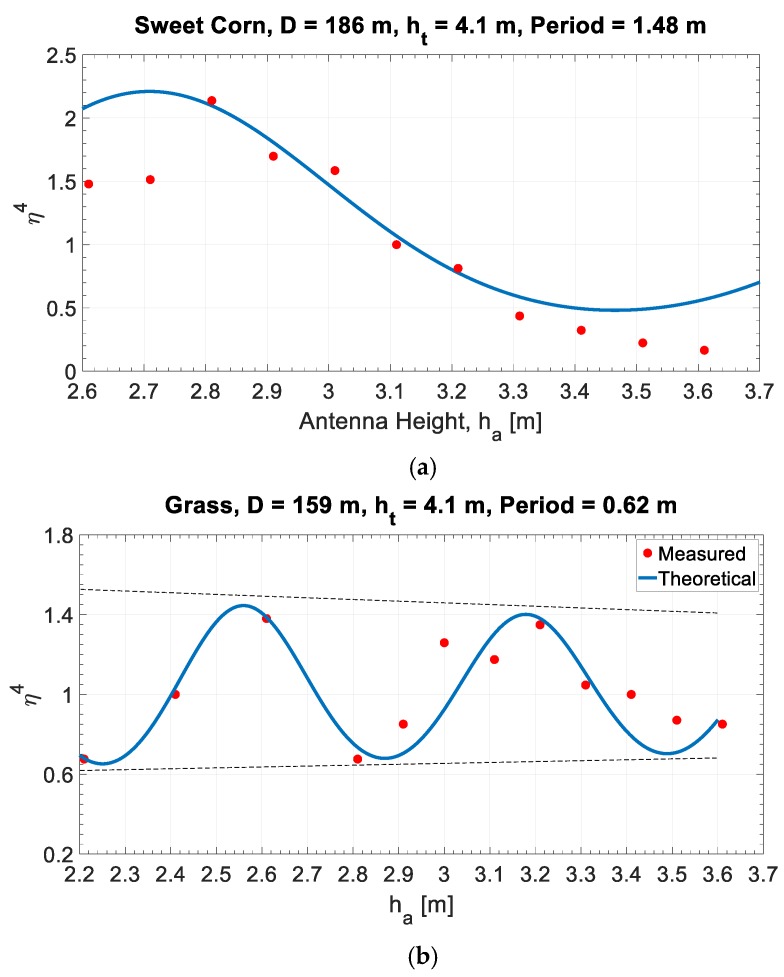
Measurement results. The period of the multipath effect is lower in propagation over grass (**b**) than over sweetcorn (**a**), due to the different height of the two kinds of vegetation.

Figure 8 shows the measured multipath effect on the RCS measurement of the standard target.

In both graphs of Figure 8 the periodic multipath effect is clearly seen. The measured values (red dots) are in good agreement with the theoretical values drawn as a blue line. The influence on multipath of the different kinds of vegetation can be easily read from the graphs. The amplitude of the oscillation is higher in the case of propagation over sweetcorn while an oscillation with lower amplitude but higher frequency is observed over grassland.

These results represent a single snapshot of a single day without any significant variation in temperature, humidity and more; anyway, this single comparison between two different kinds of vegetation results in significant observations. Long-term measurements are presented in the ensuing section describing the variations of the multipath effect over time.

## 6. Long-Term Measurements of Radar Cross Section of Man-Made Targets

**Figure 9:**
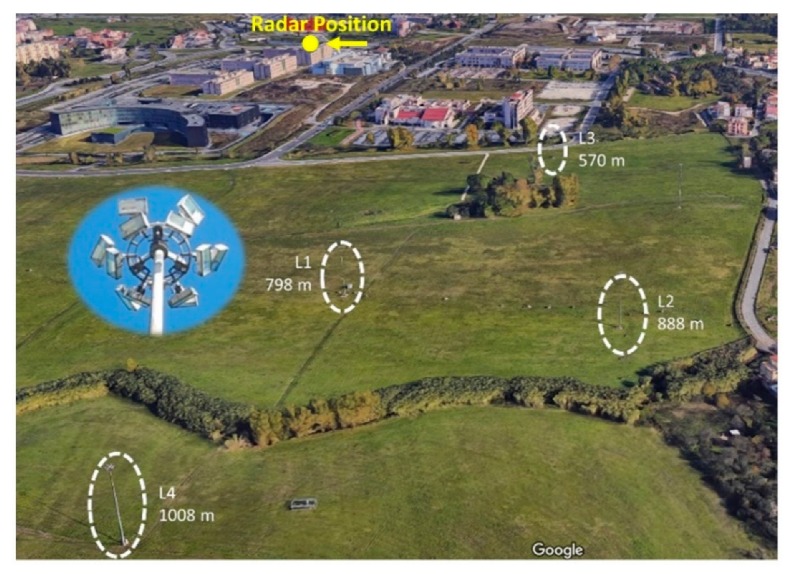
Overview (from Google Earth) of the trials environment at the Tor Vergata University area. The four opportunity targets (lampposts L1, L2, L3, and L4) are highlighted.

In the frame of the 2018 experiments at Tor Vergata campus and their 2019 follow-on, fixed identical and very large lampposts (30 m high, here referred to as L1, L2, L3, and L4) at different distances from the radar (798, 888, 570, and 1008 m, respectively) were considered as opportunity targets. Figure 9 displays an overview of the area, showing the position of the radar and of the lampposts.

**Figure 10:**
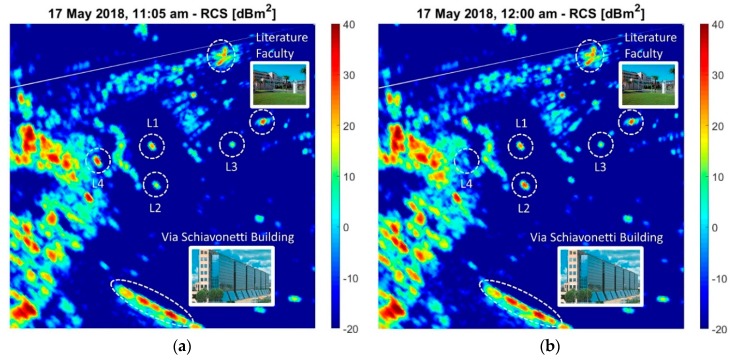
Measured RCS, 17 May 2018: (**a**) 11:05 and (**b**) 12:00.

Each lamppost has six pairs of large lamps arranged on a metallic ring, as shown in the inset of Figure 9. As the top of each lamppost is similar to a single, small, but strong radar scatterer at 30 m above ground level, the radar echo from these targets are candidates to be significantly affected by multipaths. Using Equation (3) and the (Range, Azimuth) matrix of the ADC levels, an RCS map of the environment has been generated, as shown in Figure 10. In these measurement results, two significant target echoes are created by the compound of the Literature Faculty and the long commercial building in via Schiavonetti.

Unexpectedly, during the trials on 17 May 2018, the estimated values of the RCS, in two close times of this day (11:05 and 12:00) have shown a considerable difference for lampposts L2 and L4. For L2, the measured RCS changed from $19.1{\;\mathrm{dBm}}^{2}$ to $32.4{\;\mathrm{dBm}}^{2}$, while for L4, it changed from $33.8{\;\mathrm{dBm}}^{2}$ to $8.1{\;\mathrm{dBm}}^{2}$ (see Figure 10a,b, where the RCS in ${\mathrm{dBm}}^{2}$ is shown as a false color in an area including the four lampposts).

The rationale for these significant variations can only be attributed to the propagation effects, in particular, to some time-varying multipath. In fact, in some experiments, we found that the reflecting surface on the ground is characterized by many time-varying scatterers (such as vegetation, foliage of trees, tall grass, etc.), and we know that the multipath can significantly affect the measurements of the RCS.

The sensitivity of the measured RCS w.r.t. the height of the target (see Appendix A) increases significantly when this height (in wavelengths) increases. Hence, for a tall scatterer such as a building (e.g., in Figure 10, the building at Via Schiavonetti, and the ones of the Faculty of Literature), the multipath effects average along the elevation, and, thus, the measured RCS is stable; the opposite is true for a point target, such as a small flying object or, neglecting the contribution by its pole, a lamppost at a fixed height. Therefore, for large and tall targets and for most buildings, the measured RCS has steady values as the multipath effect vanishes ($\eta_{dB}^{4}≅0\;\mathrm{dB}$).

**Figure 11:**
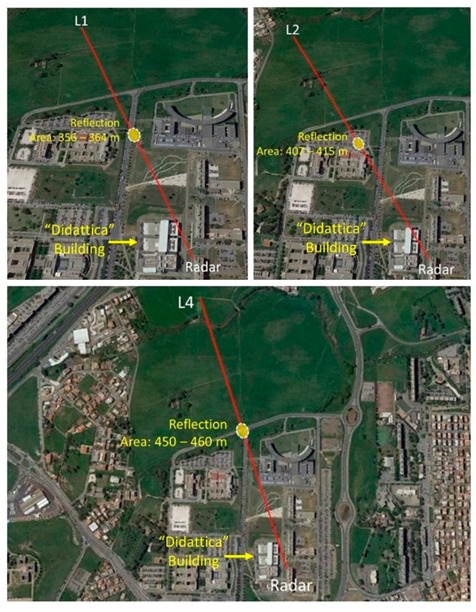
Reflection points on the ground for L1, L2, and L4.

**Figure A1:**
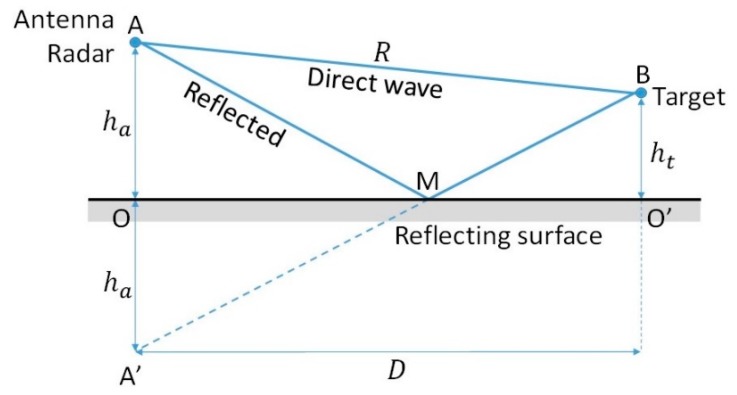
Geometry of the radar propagation on a plane-reflecting surface.

To better understand this point, more measurements of the four lampposts were carried out in spring 2019, varying the antenna height. Five 25 cm-step positions, from a maximum elevation (position 1) to a minimum elevation (position 5), were used for radar data acquisition. Figure 11 shows the reflection points (see point M in Figure A1) on the ground for lampposts L1, L2, and L4.

**Figure 12:**
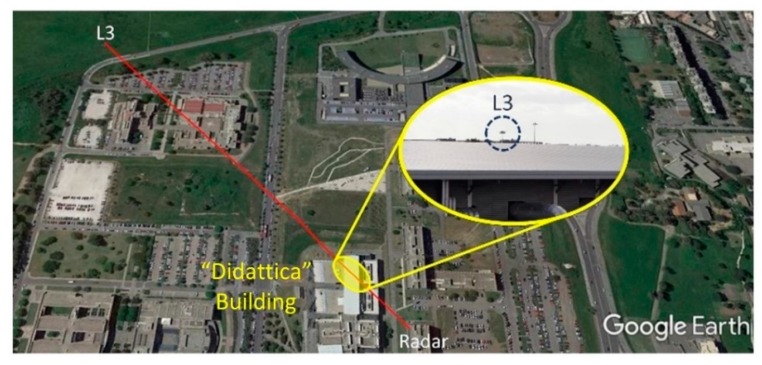
Reflection point on the ground for L3.

For lampposts L1 and L4, the reflection points shifted on the road, by 7−8 m, due to the variation of the antenna height. Hence, the reflection points’ positions may move from vegetated land into a road, possibly with some traffic. For the lamppost L2, the reflection point is on the parking lot of the Faculty of Literature, and the multipath may change rapidly if cars or buses move or appear/disappear during the acquisition. This explains the noticed, strong variations in RCS. The pole of the lamppost L3 is partially masked by the roof of the “Didattica” building (see Figure 12), hence, multipath effects are considered negligible for the RCS measurements of L3.

**Figure 13:**
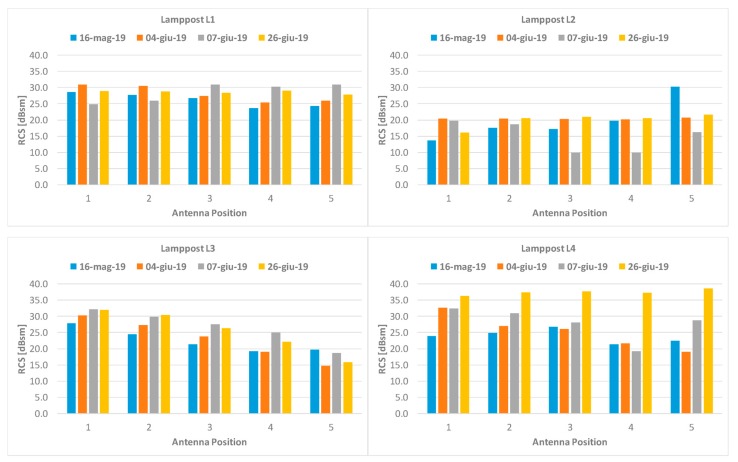
Measured RCS (dBsm) of the lampposts L1, L2, L3, and L4 with the antenna position varying (one step 0.25 m).

We collected the measurements obtained over the following four different days: 16 May 2019, 4 June 2019, 7 June 2019, and 26 June 2019. Figure 13 shows the measured RCS (in dB square meter) of these four lampposts, with a varying antenna elevation (five steps, 0.25 m each). 

**Figure 14:**
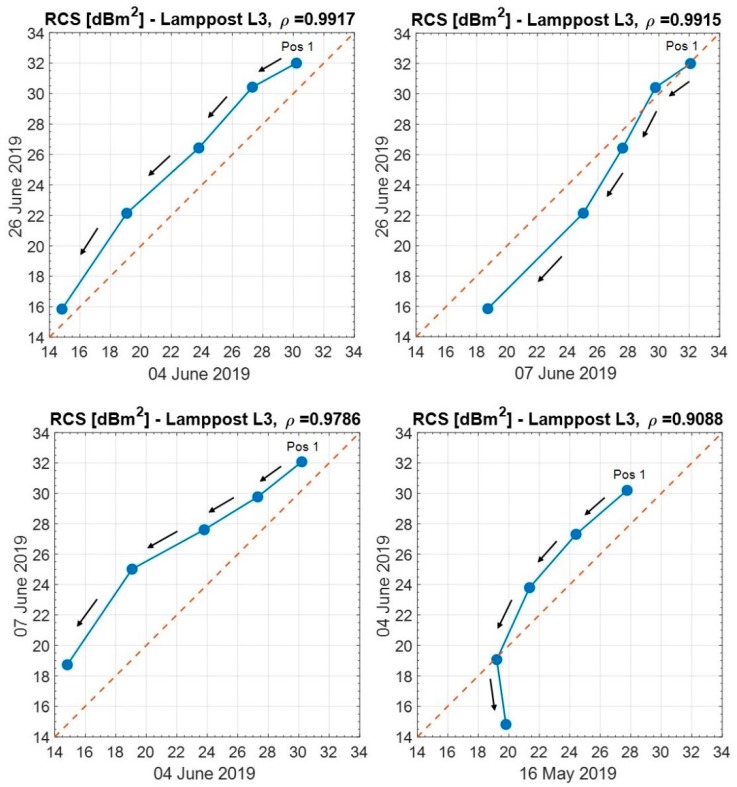
Comparison of the measured RCS of lamppost L3 on different days, varying the antenna position. Starting from the maximum antenna elevation (Position 1), the arrows denote the successive positions of the antenna up to the minimum elevation.

Lamppost L3, which is the reflection-masked target practically unaffected by the multipath effects, shows the reflection behavior different from the remaining three lampposts. During the different days, the RCS of the lamppost L3 always decreases from a maximum (step 1) to a minimum (step 5), as shown in Figure 14, due to an increased occlusion by the “Didattica” building. The temporal variation of the multipath effect is described by the parameter ρ, which has been evaluated as follows:

(6) $$\rho =\frac{\sum_{i=1}^{n}\left(X_{i}-\bar{X}\right)\left(Y_{i}-\bar{Y}\right)}{\sqrt{\sum_{i=1}^{n}{\left(X_{i}-\bar{X}\right)}^{2}\sum_{j=1}^{n}{\left(Y_{j}-\bar{Y}\right)}^{2}}}$$

where n=5 denotes the number of positions for each day, while $\bar{X}=\frac{1}{n}\overset{n}{\underset{i=1}{\sum }}X_{i}$ and $\bar{Y}=\frac{1}{n}\overset{n}{\underset{i=1}{\sum }}Y_{i}$ are the sample means.

The measurement of the RCS of lamppost L3 does vary a little from day to day, making the correlation coefficient ρ of the measured RCS on two different days (X,Y) close to the unit (Figure 14).

A different situation arises for the lampposts L1, L2, and L4, where the measured RCSs (at the same position on different days) show differences that can reach 10 dB or more. These significant variations must be attributed to the propagation effects, that is, to a time-varying multipath.

**Figure 15:**
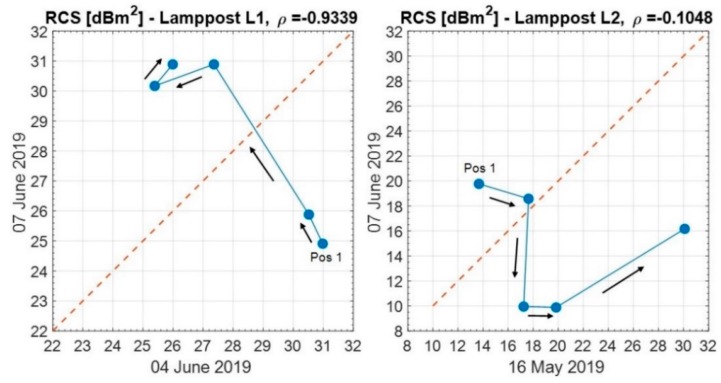
Comparison of the measured RCS of lampposts L1 and L2 on different days, varying the antenna position. Starting from the maximum antenna elevation (Position 1), the arrows denote the successive positions of the antenna up to the minimum antenna elevation.

For lamppost L1 and L2, Figure 15 shows a comparison of the measured RCS, on different days, varying the antenna position.

For L1, the values show a negative correlation (ρ=−0.9339), while for L2, they are quite de-correlated (ρ=−0.1048). These results are strongly affected by the time variation of the reflector coefficient (moving vehicles on the road, day-to-day variation, etc.)

## 7. Comments and Conclusions

Field experiments have been performed in suburban and country environments to better understand radar propagation and scattering effects, with particular attention to multipath. To this aim, it has been necessary to calibrate the radar. In general, radar calibration is a cumbersome task, requiring standard reflectors (such as corner reflectors and metal-coated spheres) and the compensation of the backscatter from the underlying surface (i.e., land) and of the multipath in the vertical plane (assuming the horizontal plane free from multipath). In Tor Vergata trials we used a non-coherent radar (with Doppler measurements not available) operating in the marine X-band with horizontal polarization. Some preliminary indications from the experimental results are synthesized as follows.

First, the unwanted reflection and scattering may be mitigated using clutter fences, with the calibration reflectors positioned in order to be seen from the radar “just above the fence”.

Second, the multipath (when not avoided by a fence) may strongly affect the calibration measurements, even when due to an apparently a more “benign” surface (slightly wet land, limited vegetation) that the well-known, strongly reflecting sea surface. The multipath compensation calls for estimating the reflection coefficient by a set of measurements (order of five to ten). In a not uniform environment (e.g., with different vegetation, roads, trees, etc.), it is necessary to vary both the height of the reflector and of the radar antenna, in order to keep the reflection point fixed on ground.

Furthermore, different experiments have proven that the multipath effect is independent of the kind of radar emission, that is, pulsed or continuous emission. Instead, it is the range resolution of the signals that is important. Future investigations might determine the influence of the radar bandwidth on the RCS variations created by the multipath effect.

As a side effect of the calibration procedure after multipath compensation, a significant “bonus”, which is obtained, is the “black box” evaluation of the radar equipment total losses.

## Figures and Tables

**Table 1:** Main parameters of the Navico/Simrad SRT radar.

Power transmitter (cavity magnetron): $P_{t}=12\;\mathrm{kW}$ (nominal) Operating frequency: f=9410 MHz (λ=0.032 m)
Transmitted pulse length: Short range mode 50 ns, medium range mode 250 ns, and long range mode 800 ns
Pulse repetition frequency (PRF): Short range mode 3000 Hz, medium range mode 1500 Hz, and long range mode 750 Hz
Antenna type: 6–feet (1.8 m) H-polarized slotted waveguide Maximum gain: $G_{dB}=29\;\mathrm{dB}$
Elevation beam-width: 25° Horizontal beam-width: 1.35°
Revolution speed: 24−48 RPM (revolutions per minute), selectable
Receiver: logarithmic with 95 dB dynamic range Noise figure: F=5 dB (nominal)
Analog-to-digital-converter (ADC): 8 bit @ 50 Msample/s

**Table 2:** Five positions during the trials on 16 May 2019. The last column shows the radar cross section (RCS) estimate for the medium corner reflector (CR).

Position	$\Delta_{a}\;\left[cm\right]$	$\Delta_{t}\;\left[cm\right]$	Time	$\mathrm{RCS}\;[\mathrm{dB}m^{2}]$
**1**	0	0	10:45	12.1
**2**	−25	−5	10:55	9.1
**3**	−50	−10	11:07	8.3
**4**	−75	−15	11:20	6.0
**5**	−95	−19	11:35	6.0

## Appendix A. The Phenomenon of Multipath

Let M be the point of reflection on the ground (Figure A1). The ground distance y=OM of the point from the radar is a fraction of the distance $D=OO^{\prime }$ of the target, as follows:

(A1) $$y=\frac{h_{a}}{h_{a}+h_{t}}D$$

For smooth flat Earth, $h_{a}$ and $h_{t}$ are the heights of the radar antenna and of the target above the land surface, respectively. The antenna pattern is considered to be uniform in the elevation angle. R denotes the distance (direct path AB) between the radar and the target (Figure A1). The change of the field strength $\left|\vec{E}\right|$ (V/m) at the target due to the multipath is expressed by the following ratio [4]:

(A2) $$\eta =\frac{\left|\vec{E}\right|}{\left|{\vec{E}}_{0}\right|}$$

where $\left|\vec{E}\right|$ is the amplitude of the field at the target in the presence of the Earth’s surface, and $\left|{\vec{E}}_{0}\right|$ is the amplitude of the target in free space. In the literature, the coefficient η is also known as the “propagation factor” F when it includes the effects of the antenna pattern on the elevation coverage, [4].

The length difference between the direct (AB) ray and the reflected one (AMB=A′MB) causes a phase difference between both signals, hence affecting their coherent sum. The differences in the amplitude and in the phase of the direct and reflected signals depend on the complex reflection coefficient, namely: $\Gamma =\rho e^{\psi_{r}}$. From Figure A1, with the approximation of $\sqrt{1+x^{2}}≅1+\frac{x^{2}}{2}$ for x≪1 that is, where $\left|h_{a}-h_{t}\right|\ll D$, as in our experiments, the path difference is approximated as follows:

(A3) $$\Delta =D\left[\sqrt{1+\frac{{\left(h_{a}+h_{t}\right)}^{2}}{D^{2}}}-\sqrt{1+\frac{{\left(h_{a}-h_{t}\right)}^{2}}{D^{2}}}\right]≅\frac{2h_{a}h_{t}}{D}.$$

The sum signal is ${\bar{X}}_{sum}={\bar{X}}_{dir}\left[1+\rho e^{j\left(\psi_{r}+\psi_{\Delta }\right)}\right]$, with $\psi_{\Delta }=\frac{2\pi }{\lambda }\Delta$. Hence,

(A4) $$\left|\frac{{\bar{X}}_{sum}}{{\bar{X}}_{dir}}\right|=\left|1+\rho e^{j\left(\psi_{r}+\psi_{\Delta }\right)}\right|\overset{\mathrm{def}}{=}\eta .$$

The multipath factor is the fourth power of η, as follows:

(A5) $$\eta^{4}={\left[1+2\rho \cdot cos\left(\psi_{r}+\psi_{\Delta }\right)+\rho^{2}\right]}^{2}.$$

For low grazing angles, that is, with $\psi_{r}≅\pi$, where $\psi_{\Delta }≅\frac{2\pi }{\lambda }\frac{2h_{a}h_{t}}{D}$, we have the following:

(A6) $$\eta^{4}={\left[{\left(1-\rho \right)}^{2}+4\rho \cdot sin^{2}\left(\frac{2\pi }{\lambda }\frac{h_{a}h_{t}}{D}\right)\right]}^{2}.$$

The maximum of $\eta^{4}$ is $\eta_{max}^{4}={\left(1+\rho \right)}^{4}$, while the minimum is $\eta_{min}^{4}={\left(1-\rho \right)}^{4}$. Hence, the variation in the “apparent” radar cross section of the target is (in decibel) as follows:

(A7) $$\Delta RCS\;\left[dB\right]=10\cdot log_{10}\left(\frac{\eta_{max}^{4}}{\eta_{min}^{4}}\right)=40\cdot log_{10}\left(\frac{1+\rho }{1-\rho }\right).$$

Table A1 shows some values of ΔRCS [dB] for different values of ρ.

**Table A1:** Maximum target RCS variation due to multipath.

ρ	1.0	0.8	0.7	0.6	0.5	0.3	0.2	0.1
$\Delta RCS_{dB}$	+∞	38.1	30.1	24.1	19.1	10.8	7.0	3.5

The multipath factor, Equation (A6), is a periodic function. A full cycle is achieved when $\frac{2\pi }{\lambda }\frac{h_{a}h_{t}}{D}≐\pi$, that is, $h_{a}h_{t}=\frac{\lambda D}{2}$. Hence, setting $h_{a}$ while varying $h_{t}$ (typical experimental set-up with standard calibrated targets, such as corner reflectors and metallized spheres), the period is $\frac{\lambda D}{2h_{a}}$. On the other hand, setting $h_{t}$ (typical experiments using large and heavy opportunity targets such as lampposts, buildings, towers, and tanks) and varying $h_{a}$, the period is $\frac{\lambda D}{2h_{t}}$. For the latter case, Table A2 shows the period of $\eta^{4}$ for λ=0.03 m, D=1000 m, and various heights of the target.

**Table A2:** Period of $\eta^{4}$ for λ=0.03 m and D=1000 m.

$h_{t}\;\left[m\right]$	2.0	5.0	10.0	15.0	20.0	30.0
$\frac{\lambda D}{2h_{t}}$ [m]	7.5	3.0	1.5	1.0	0.75	0.5

For ρ=1 ($\psi_{r}=\pi$), Equation (A6) becomes the following:

(A8) $$\eta^{4}=16\cdot sin^{4}\left(\frac{\pi \Delta }{\lambda }\right)≅16\cdot sin^{4}\left(\frac{2\pi }{\lambda }\cdot \frac{h_{a}h_{t}}{D}\right)$$

The theoretical range for $\eta^{4}$ in a linear scale is [0, 16], and in decibels is (−∞, 12]. From Equation (A1), posing $r=\frac{h_{a}}{h_{t}}$, the sensitivity w.r.t. $h_{a}$ (resp. $h_{t}$), is easily computed as follows:

(A9) $$\begin{array}{l} S_{h_{a}}^{y}=\frac{h_{a}}{y}\cdot \frac{dy}{dh_{a}}=\frac{h_{t}}{h_{a}+h_{t}}=\frac{1}{1+r}\\ S_{h_{t}}^{y}=\frac{h_{t}}{y}\cdot \frac{dy}{dh_{t}}=-\frac{1}{1+r} \end{array}.$$

When both heights are allowed to vary, the overall percentage variations of the reflection point distance OM (Figure A1) are as follows:

(A10) $$\Delta y\left(\%\right)≅\Delta h_{a}\left(\%\right)\frac{1}{1+r}-\Delta h_{t}\left(\%\right)\frac{1}{1+r}.$$

In order to maintain the reflection point fixed on the ground (i.e., ensure that Δy=0), equal percentage variations shall be applied to both $h_{a}$ and $h_{t}$, according to Equation (A10).
